# Association between Bone Mineral Density and Metabolic Syndrome among Reproductive, Menopausal Transition, and Postmenopausal Women

**DOI:** 10.3390/jcm10214819

**Published:** 2021-10-20

**Authors:** Rogelio Salas, Alexandra Tijerina, Mariana Cardona, Cristina Bouzas, Erik Ramirez, Gustavo Martínez, Aurora Garza, Rosario Pastor, Josep A. Tur

**Affiliations:** 1 Faculty of Public Health and Nutrition, Autonomous University of Nuevo Leon, Monterrey 64460, NL, Mexico; rogersalas1@hotmail.com (R.S.); alexandra.tijerinas@uanl.mx (A.T.); erik.rale@gmail.com (E.R.); bioestadistica@hotmail.com (G.M.); 2 Faculty of Nursing and Nutrition, Autonomous University of Chihuahua, Chihuahua 31110, CH, Mexico; macardona@uach.mx; 3 Research Group on Community Nutrition and Oxidative Stress, University of Balearic Islands-IUNICS & IDISBA, 07122 Palma de Mallorca, Spain; cristina.bouzas@uib.es (C.B.); rosario.pastor@ucavila.es (R.P.); 4 CIBER Physiopathology of Obesity and Nutrition (CIBEROBN), Institute of Health Carlos III (ISCIII), 28029 Madrid, Spain; 5 Faculty of Medicine, Autonomous University of Nuevo Leon, Monterrey 64460, NL, Mexico; aurgarza@gmail.com; 6 Faculty of Health Sciences, Catholic University of Avila, 05005 Avila, Spain

**Keywords:** menopausal transition, postmenopausal, metabolic syndrome, bone mineral density, women, Mexico

## Abstract

The menopausal transition stage brings physiological changes associated with the development of metabolic syndrome (MetS), which can affect bone mineral density (BMD), and may be more evident in the postmenopausal stage. The aim of this study was assessing the association between low BMD and MetS and its components among reproductive/menopausal transition and postmenopausal women in the northeast region of Mexico. A descriptive cross-sectional study was carried out (2015–2016) in 40–60-year-old women (*n* = 376) who were residents in the metropolitan area of Monterrey, in Nuevo Leon State, Mexico. Anthropometric measurements, blood pressure, a dual-energy X-ray absorptiometry (DXA) evaluation of BMD of two anatomical sites (lumbar spine and dual femur), and a biochemical analysis were obtained. The prevalence of MetS was 57.2%. In participants without MetS, the prevalence of osteopenia was 27.3% in the lumbar spine and 18.6% in the dual femur, while in participants with MetS, the prevalence of osteopenia was 35.8% in the lumbar spine and 14.4% in the dual femur. Osteoporosis in participants without MetS was present in 6.8% in the lumbar spine and in 1.8% in the dual femur, while in women with MetS, its prevalence was 4.7% in the lumbar spine and 0.5% in the dual femur. An association between low BMD at the lumbar spine and dual femur and components of MetS diseases was identified in Mexican women as follows: waist circumference ≥ 88 cm showed an increase risk for low BMD at femoral site in both reproductive/menopausal transition (OR 7.638; 95% CI: 1.607–36.298; *p* = 0.011) and postmenopausal women (OR 2.600; 95% CI: 1.023–6.609; *p* = 0.045); HDL < 50 mg/dL was associated with low BMD in both the femur (OR 3.639; 95% CI: 1.039–12.743; *p* = 0.043) and lumbar spine (OR 2.654; 95% CI: 1.092–6.447; *p* = 0.031); hypertension in postmenopausal women increased the risk for low BMD in the femur (OR 2.634; 95% CI: 1.150–6.035; *p* = 0.022). In conclusion, we found that components of the MetS were associated with low BMD, thus indicating that MetS increases the risk for developing osteopenia or osteoporosis. Furthermore, age was found to be an independent risk factor for low BMD.

## 1. Introduction

Early and late menopausal transition and postmenopausal periods include biological, endocrinological, clinical, and psychological events, and bring physiological changes associated with the development of metabolic syndrome (MetS) [1,2]. MetS is defined as the cluster of biochemical, physiological, and anthropometric abnormalities that occur simultaneously and are linked with insulin resistance, and it increases the risk of developing noncommunicable diseases such as type 2 diabetes mellitus, cardiovascular disease, or both [3]. The etiology of MetS is multifactorial and complex; however, its main predictable components are abdominal obesity and insulin resistance [4].

MetS components have been related to decreased bone mass throughout ovarian hormone changes, mainly during the menopausal transition, which alters other mechanisms including body composition as abdominal fat increases as well as altered lipid and glucose metabolism [5,6]. Hyperglycemia and increased adipose tissue have been associated with a poor skeletal health as the altered insulin signaling may be related to a reduction of bone formation [7], and consequently a decrease in bone mineral density (BMD), hence developing osteopenia and osteoporosis [5,8]. Low HDL levels and an inflammatory microenvironment affect the differentiation and function of osteoblasts [9]. In addition, it has been reported that dyslipidemia [10] and high blood pressure [11] are risk factors for low bone mass.

In Mexico, there is a high prevalence of overweight, obesity, and MetS in women. According to the Mexican National Survey on Health and Nutrition (*Encuesta Nacional de Salud y Nutricion*, ENSANUT) in 2018–2019, the prevalence of overweight and obesity among Mexican women was 76.8%, with higher rates in the Northern States. The criteria of MetS with highest prevalence were abdominal obesity, identified as 92.1% in 40–49-year-old women and 95.8% in 50–59-year-old women [12]. A descriptive study in Nuevo Leon State, Mexico, reported a prevalence of MetS of 59.4% in ≥16-year-old women, and an increased prevalence of components of MetS with increasing age, except for HDL levels, showing an important public health problem [13]. A systematic review pointed out that the prevalence of MetS in Mexican adult women was 41% (95% CI 0.34–0.47) [14]. Osteopenia and osteoporosis in various states in Mexican adult women have been reported from 30.12% to 39.8% and 13.6%, respectively [15,16]; however, there is limited evidence in the region of the Nuevo Leon State.

The association of BMD with MetS and its components has not been concluded yet [17,18]. Studies have pointed out a protective effect on bone health due to the presence of MetS, obesity, and diabetes [19,20], but several others have reported an increased risk of osteopenia or osteoporosis in individuals with diabetes, high blood pressure, and hypertriglyceridemia [17,21,22,23,24]. In addition, there is limited scientific evidence from Mexican women, specifically in menopausal transition and postmenopausal stage.

The aim of this study was assessing the association between low BMD and MetS and its components among reproductive/menopausal transition and postmenopausal women in the northeast region of Mexico.

## 2. Methods

### 2.1. Design

A descriptive cross-sectional study was carried out (2015–2016) in 40–60-year-old women residents in the metropolitan area of Monterrey, in Nuevo Leon State, Mexico. A sample size of *n* = 376 was determined as representative according to the finite population equation with 5% level of precision, a level of confidence interval of 95%, a 40–60-year-old women population of 491,024, and the prevalence of MetS at 59.4% [13].

Women were invited to participate via social media, telephone calls, and flyers posted on medical centers and public areas, and voluntary response sampling was followed. Exclusion criteria were abandonment of the study, incomplete data from the subject, and pregnancy. Women using metallic artifacts in the spine or femoral sites and/or a cardiac pacemaker were also excluded as image results from DXA are affected by these [25]. The study was developed following the Declaration of Helsinki. It was approved by the Ethics Committee of the Faculty of Public Health and Nutrition of the Autonomous University of Nuevo León (protocol ID: 15-FaSPyN-SA-11). Written informed consent was obtained from each participant. Data collection was performed in the Centre for Research in Nutrition and Public Health of the Faculty of Public Health and Nutrition.

### 2.2. Clinical History

Complete clinical history included information on the participant’s date of birth, day of last menses, changes in menstrual cycles, number of children, use of drugs, smoking habit (current smoker: yes/no), presence of metallic artifacts, and use of a cardiac pacemaker. Menstrual cycles and changes were identified, and women were grouped as follows: reproductive, as regular cycles or not noticeable changes were present (*n* = 55); menopausal transition, as an altered duration in cycles of ≥7 days or intervals of amenorrhea of ≥60 days (*n* = 91); and postmenopausal, as the absence of a menstrual cycle for ≥12 months (*n* = 230), according to STRAW+10 criteria [26]. Women were classified in group 1 (reproductive and menopausal transition), and group 2 (postmenopausal).

### 2.3. Metabolic Syndrome Definition

MetS was defined according to the census definition of IDF/NHLBI/AHA/WHF/IAS/IASO [1], by meeting three or more of the following components: (a) waist circumference (WC) ≥80 cm, (b) serum triglycerides ≥ 150 mg/dL, (c) serum HDL < 50 mg/dL, (d) blood pressure ≥ 130/85 mmHg or antihypertensive medication, and (e) fasting serum glucose ≥ 100 mg/dL or diabetic treatment. WC ≥ 88 cm was also considered in the analysis, as defined by the Adult Treatment Panel III (ATP-III) as a clinical risk factor to metabolic syndrome [27].

### 2.4. Anthropometric Measurements

Height was determined to the nearest millimeter using a digital stadiometer (SECA 274, Hamburg, Germany), with the subject’s head in the Frankfurt plane. Body weight (kg) was determined to the nearest 100 g by bioelectrical impedance analysis (Inbody A120 & Software Lookin’Body 120, Inbody Co., Seoul, Korea), which also provided with the body mass index (BMI, kg/m^2^), which was classified as normal weight, overweight, and obese according to NORMA Oficial Mexicana NOM-008-SSA3-2017 [28]. Waist circumference (WC) was measured to the nearest 0.1 cm using a non-stretch measuring tape (SECA 201, Hamburg, Germany) at the midpoint between the last rib and the iliac crest.

### 2.5. Blood Pressure Measurements

Blood pressure (BP) measurements were performed to the nearest 1 mmHg and were taken using a Beurer blood pressure monitor (BM19, Beurer Medical instruments, Ulm, Germany) with subjects seated in a resting chair using the left arm with the palm facing upward, according to NORMA Oficial Mexicana NOM-030-SSA2-2009 [29]. Two readings were taken 5 min apart and the average was taken.

### 2.6. Biochemical Assays

Venous blood samples were obtained from the antecubital vein in suitable vacutainers after 12 h overnight fasting, as established by the NORMA Oficial Mexicana NOM-253-SSA1-2012 [30]. Blood samples were centrifuged at 3500 rpm for 12 min. Serum was frozen at −80 °C until assays were performed. Biochemical assays were performed in the A25 automatic analyzer, software v4.1.1, and commercial kits to determine glucose (CV = 1.2%), triglycerides (CV = 1.6%), and HDL cholesterol (CV = 0.8%) (BioSystems^®^ S.A, Barcelona, Spain).

### 2.7. Bone Mineral Density

BMD was measured using a dual-energy X-ray absorptiometry (DXA) equipment (Lunar *i*DXA and enCORE software version 16; GE Healthcare, Madison, WI, USA) in two anatomical sites: the anteroposterior (AP) lumbar spine and the dual femur (CV = 1.0%). Before starting the measurements, the equipment was calibrated according to the instructions in the manual regarding quality control. Measurements were conducted after a 4 h of fasting. Subjects were classified as normal, osteopenia, or osteoporosis according to the WHO diagnostic criteria [31]. T-score values were automatically determined by enCORE software after measured participants’ BMDs were compared to a reference BMD from the female young-adult population. For a normal BMD, T-scores were equal to or above −1.0, osteopenia T-scores were above −2.5 and below −1.0, and osteoporosis was defined as a T-score equal to or below −2.5 [31].

### 2.8. Statistical Analysis

Data were analyzed for normality by Kolmogorov–Smirnov test. Participants were stratified according to the defined groups (group 1: reproductive/menopausal transition women, and group 2: postmenopausal women) and by the presence or absence of MetS. Differences between groups for numerical variables were calculated by the Student’s *t*-test. Differences between groups in categorical variables were analyzed by the Chi-squared test.

A multivariate logistic regression was used to assess the association between low BMD in the dual femur and in the spine (dependent variable coded as osteopenia/osteoporosis: yes/no) and the following risk factors—WC, glucose, triglycerides, HDL, systolic blood pressure, diastolic blood pressure, age, BMI, BMI ≥ 25 kg/m^2^ smoking, and number of children (independent variables)—in no MetS and MetS women, as well as in defined groups 1 and 2.

Another multivariate logistic regression was used to assess the association between low BMD (dependent variable coded as osteopenia/osteoporosis: yes/no) and components of MetS applying definitions of IDF/NHLBI/AHA/WHF/IAS/IASO [1] and the Adult Treatment Panel III (ATP-III) as a clinical risk factor to metabolic syndrome [27] (independent variable) in groups 1 and 2, which were adjusted by the presence of MetS, as well as age, smoking, and number of children as risk factors for osteoporosis [32,33,34] as covariates.

Analyses were performed in SPSS statistical software package (SPSS v.21 for Windows, IBM Software Group, Chicago, IL, USA) and significance was set at *p* < 0.05.

## 3. Results

Table 1 shows characteristics of participants stratified by presence of absence of MetS.

The prevalence of MetS was 57.2%, and postmenopausal stage (group 2) was predominant in those women with MetS (66.0%). The age was 50.7 ± 5.4 years in women with MetS and 49.6 7 ± 5.4 years in women without MetS (*p* = 0.035). A normal BMI was mainly in women without MetS (41.6%), and obesity predominated in those with MetS (56.7%). BMD in the dual femur was 1.02 ± 0.13 g/cm^2^ in women with MetS and 0.97 ± 0.13 g/cm^2^ in women without MetS (*p* < 0.001), but there were no significant differences in the lumbar spine BMD (*p* = 0.660). In participants without MetS, the prevalence of osteopenia was 27.3% in the lumbar spine and 18.6% in the dual femur, while in participants with MetS, the prevalence of osteopenia was 35.8% in the lumbar spine and 14.4% in the dual femur. The prevalence of osteopenia in the spine was higher in women with MetS when compared to women without MetS, but the result was not significant (*p* = 0.054). In participants without MetS, osteoporosis was present in 6.8% in the lumbar spine and in 1.8% in the dual femur, while in women with MetS, its prevalence was 4.7% in the lumbar spine and 0.5% in the dual femur. Significant differences (*p* < 0.001) occurred in all components of MetS, as expected. The mean WC was higher than 85 cm in both groups, 98.1 ± 12.0 cm in women with MetS and 85.0 ± 11.9 cm in women without MetS. Mean HDL levels were lower than 50 mg/dL in both groups, 34.5 ± 9.2 mg/dL in women with MetS and 40.5 ± 13.7 mg/dL in women without MetS. Medical treatment use was higher in women with MetS (*p* < 0.001). Antihypertensive treatment was used in 20.9% of women with MetS, but only in 1.9% of women without MetS. There was no difference between groups in the number of children and current smoking habit.

Results from a multivariate logistic regression analysis for low BMD in the dual femur (Table 2) and spine (Table 3), according to presence of MetS and stage, are shown. In Table 2, independent variables showed no association for low BMD in the dual femur, except for age, WC, and number of children in those women with MetS in the postmenopausal stage. Age was an independent factor that increased the risk by 29.8% for having a low BMD in the dual femur (95% CI: 1.109–1.520, *p* = 0.001) in postmenopausal women. WC reduced the risk by 7.5% for having low BMD in the dual femur in postmenopausal women with MetS (95% CI: 0.857–0.999, *p* = 0.047). The number of children reduced the risk of low BMD in the dual femur in postmenopausal women with MetS (95% CI: 0.456–0.997, *p* = 0.048).

Low BMD in the spine L1–L4 was mainly associated, in women with MetS, with WC, glucose level, age, BMI, smoking, and number of children. There was a reduced risk for presenting low BMD in the spine shown by WC (OR 0.790; 95% CI: 0.635–0.98; *p* = 0.034), BMI ≥ 25 kg/m^2^ (OR 0.007; 95% CI: 0.003–0.476; *p* = 0.021), and smoking habit (OR 0.005; 95% CI 0.001–0.509; *p* = 0.025), but an increased risk by age (OR 1.309; 95% CI 1.013–1.690; *p* = 0.039) in reproductive/menopausal transition women (group 1) with MetS. However, the postmenopausal stage showed increased risk for presenting low BMD in the spine, associated to glucose (OR 1.013; 95% CI: 1.001–1.026), *p* = 0.037), HDL levels (OR 1.084; 95% CI: 1.030–1.140; *p* = 0.002), and age (OR 1.189; 95% CI: 1.070–1.322; *p* = 0.001), but lowered by the number of children (OR 0.611; 95% CI 0.431–0.867; *p* = 0.006).

To determine whether there is an association between low BMD and the components of MetS, a multivariate binary logistic regression analysis was performed, adjusting for the presence of metabolic syndrome, age, smoking habit, and number of children, in both reproductive/menopausal transition (group 1) and postmenopausal stage (group 2) women (Table 4). Waist circumference ≥88 cm showed an increased risk for low BMD at the femoral site in both reproductive/menopausal transition (group 1) (OR 7.638, (95% CI: 1.607–36.298), *p* = 0.011) and postmenopausal stage (group 2) (OR 2.600 (95% CI: 1.023–6.609), *p* = 0.045) women. There was no association with WC ≥80 cm. The component HDL at levels below 50 mg/dL was associated with low BMD in both the femur and lumbar spine, OR 3.639 (95% CI: 1.039–12.743; *p* = 0.043) and OR 2.654 (95% CI: 1.092–6.447; *p* = 0.031), respectively, but results were not consistent for women in both reproductive/menopausal transition (group 1) and postmenopausal stage (group 2). Hypertension or its treatment was associated to an increased risk of low BMD in the femur (OR 2.634 (95% CI: 1.150–6.035), *p* = 0.022) but only significant in postmenopausal women. Triglycerides were not associated to BMD.

## 4. Discussion

A cross-sectional study on reproductive/menopausal transition and postmenopausal women from the Nuevo Leon State determined significant differences among clinical and metabolic parameters when stratified according to the presence of MetS. The prevalence of MetS in 40–60-year-old Mexican women found in the current study was 57.2%, like previously reported findings (59.4%) [13]. Although there are no data respecting the worldwide prevalence of MetS, it is estimated that a quarter of the population suffers from it; therefore, around one billion people worldwide are affected by MetS [32]. Mexico has a significantly higher prevalence of MetS compared to other countries, especially in adult women, such as Portugal (45.7%), the United States (35.6%), France (15%), Spain (26.6%), and Iran (31%) [35,36,37,38].

Like other countries, the prevalence of MetS in Mexico is higher among adult women than in adult men, which may be attributed to the increase in abdominal obesity during the menopausal transition [39]. In the current study, 215 subjects were classified with MetS, 34% in reproductive/menopausal transition and 66% were postmenopausal. As women age, there was a progression on reproductive senescence [26] characterized by a decrease in ovarian hormones, which promotes central obesity, altered lipid and glucose metabolism, and hypertension [6,39], suggesting the reproductive stage as an independent factor to be further investigated. Current results from the multivariate logistic regression analyses suggested age as an independent risk factor of low BMD in the dual femur and spine for those women presenting MetS, which agrees with previous studies that attributed the effects of age to low bone mass in women [19,20,22,23,40,41,42].

A total of 90.7% of the participants with MetS were overweight or obese, a higher prevalence than previous findings among 16–60-year-old Nuevo Leon inhabitants (60.3%) [12,13]. Despite this tendency, a BMI ≥ 25 kg/m^2^ in women in reproductive/menopausal transition stages, showed a protective role to have low BMD in the spine (OR 0.007 (95% CI:0.003–0.476; *p* = 0.021), as previously reported in postmenopausal Pakistan women [42]. Moreover, it was shown that abdominal obesity, measured as waist circumference in centimeters, at levels of 80 cm or higher, was not associated to BMD; however, WC ≥ 80 cm is an independent factor associated to decrease BMD in the dual femur in both reproductive/menopausal transition and postmenopausal women. These results are similar to findings among menopausal women that reported abdominal obesity as positively related to BMD of the femoral neck [20,43,44,45], reducing the risk of fracture in women [39,45], and others concluded that obesity may lead to an increase in bone density because of its association with higher 17β-estradiol levels and higher mechanical loads, thus protecting bones [46,47]. A cross-sectional analysis in adults (≥20year-old) from United States of America reported higher femoral BMD associated to abdominal obesity (*p* < 0.001); however, there was not an association with MetS [48].

Fasting glucose was an independent factor increasing the risk for low BMD in the spine of postmenopausal women with MetS. Likewise, other authors have found that the increase in bone fragility can be caused by chronic hyperglycemia, which leads to the accumulation of microfractures or cortical porosity [17,24]. In this study, high-density lipoprotein was associated to low BMD, increasing the risk up to 3.6 times for a reduced BMD in both reproductive/menopausal transition and postmenopausal groups, when levels met the MetS criteria below 50 mg/dL. This may be due to the association between reduced levels of HDL and the development of an inflammatory microenvironment affecting osteoblasts’ differentiation and function, thus showing a decrease in BMD [9,49,50]. Moreover, a mechanism of hypertension-related osteoporosis has been proposed, as high blood pressure may lead to low bone turnover, while detecting low levels of osteocalcin in postmenopausal women with osteoporosis [51]. Blood pressure at levels of 130/85 mmHg or higher or use of antihypertensive treatment were associated with low BMD in the dual femur in postmenopausal women, like other findings in the same stage [11]. Since abdominal obesity, fasting hyperglycemia, low level of HDL, and hypertension are related to low BMD, and all of them are components of MetS, a relationship between MetS and low BMD may be expected. Moreover, MetS is more prevalent among postmenopausal than among reproductive/menopausal transition women. Thus, low BMD will be more prevalent among postmenopausal women, as it was demonstrated by the fact of half of postmenopausal women will have an osteoporosis-related fracture during their lives [52].

The current results showed that an association between BMD at the spine and dual femur and components of MetS diseases has been identified in 40–60-year-old Mexican women, as well as individuals with increased MetS components such as higher BMI, WC, altered glucose and lipid profiles, and hypertension. As age was identified as an independent factor of low BMD in women with and without MetS, there is a tendency for a higher prevalence of osteopenia and osteoporosis, and thus a higher risk of fractures, which may be more costly for health systems. These results should have influence on the design of preventive campaigns for bone health at early stages of women reproductive aging.

## 5. Strengths and Limitations

The main strength of the current study is that an association was found between BMD and MetS components in reproductive/menopausal transition and postmenopausal women. The first methodological limitation to be acknowledged is that causal inferences cannot be drawn due to the cross-sectional design; longitudinal cohort studies would be needed to give further information. A second limitation is the relatively small sample size, which avoids generalizing these findings to the broader community based on this study alone. Finally, it seems that there was higher percentage of postmenopausal participants compared to reproductive/menopausal transition women; however, the number of women in the latter stage were the same with and without MetS (*n* = 73 in both cases), but the number of postmenopausal women with MetS (*n* = 142) was higher than those without MetS (*n* = 88), as expected and concordant with previous evidence [26,36].

## 6. Conclusions

In conclusion, components of the MetS were associated with low BMD, thus indicating that MetS increases the risk for developing osteopenia or osteoporosis. Furthermore, age was found to be an independent risk factor for low BMD. Current findings can contribute to public health actions since they enrich the overview of high prevalence diseases in the country. Further studies are suggested to improve medical and nutritional intervention regarding MetS and osteoporosis in reproductive/menopausal transition and postmenopausal women.

## Figures and Tables

**Table 1:** Characteristics of 40–60-year-old women of Nuevo Leon State, Mexico.

Variable	No MetS *n* = 161	MetS *n* = 215	*p*
Age (years)	49.6 ± 5.4	50.7 ± 5.4	0.035
Stage			
Group 1: Reproductive/Menopausal transition (%)	45.3	34.0	1.00
Group 2: Postmenopausal (%)	54.7	66.0	<0.001
BMI (kg/m^2^)	26.8 ± 4.7	31.5 ± 5.8	<0.001
Normal (%)	41.6	9.3	
Overweight (%)	37.9	34.0	
Obesity (%)	20.5	56.7	
BMD dual femur (g/cm^2^)	0.97 ± 0.13	1.02 ± 0.13	<0.001
Normal (%)	79.5	85.1	
Osteopenia (%)	18.6	14.4	
Osteoporosis (%)	1.8	0.5	
BMD lumbar spine (g/cm^2^)	1.12 ± 0.15	1.13 ± 0.16	0.66
Normal (%)	65.8	59.5	
Osteopenia (%)	27.3	35.8	
Osteoporosis (%)	6.8	4.7	
Waist circumference (cm)	85.0 ± 11.9	98.1 ± 12.0	<0.001
Fasting glycaemia level (mg/dL)	90.1 ± 10.9	110.8 ± 46.6	<0.001
Triglyceride level (mg/dL)	110.5 ± 46.3	178.9 ± 91.7	<0.001
HDL cholesterol (mg/dL)	40.5 ± 13.7	34.5 ± 9.2	<0.001
Systolic blood pressure (mmHg)	109.3 ± 10.5	123.8 ± 15.5	<0.001
Diastolic blood pressure (mmHg)	68.8 ± 9.2	77.6 ± 11.5	<0.001
Medical Treatment			
Diabetes (%)	0.6	14.9	<0.001
Hypertension (%)	1.9	20.9	<0.001
Hypertriglyceridemia (%)	0.0	2.3	<0.001
Hypoalphalipoproteinemia (%)	1.9	9.8	<0.001
Smoking habit (%)	6.8	6.5	0.55
Number of children	2.4 ± 1.1	2.7 ± 1.3	0.05

Abbreviations: BMD: bone mineral density; BMI: body mass index; HDL: high-density lipoprotein; MetS: metabolic syndrome. Values are presented as mean ± standard deviation or as prevalences (%). Differences were calculated by Student’s *t*-test (numerical variables) or Chi-squared test (categorical variables). Significance at *p* < 0.05.

**Table 2:** Multivariate logistic regression analysis of low bone mineral density (osteopenia/osteoporosis) in the dual femur in 40–60-year-old women.

	No MetS (*n* = 161)			MetS (*n* = 215)		
	OR	95% CI	*p*	OR	95% CI	*p*
Group 1: Reproductive/Menopausal transition						
WC (cm)	0.975	0.878–1.083	0.640	0.978	0.681–1.404	0.903
Glucose (mg/dL)	1.040	0.971–1.115	0.259	0.990	0.855–1.145	0.889
Triglycerides (mg/dL)	1.004	0.990–1.019	0.556	0.993	0.965–1.022	0.655
HDL (mg/dL)	0.996	0.941–1.054	0.891	1.121	0.810–1.550	0.491
Systolic blood pressure (mmHg)	1.027	0.937–1.126	0.566	1.150	0.842–1.571	0.380
Diastolic blood pressure (mmHg)	1.023	0.912–1.148	0.697	0.965	0.695–1.340	0.833
Age (years)	1.090	0.882–1.342	0.426	1.851	0.579–5.923	0.299
BMI normal (kg/m^2^)	3.292	0.142–76.073	0.457	4.887	0.319–57.035	0.563
BMI ≥ 25 kg/m^2^	1.308	0.076–22.574	0.854	0.284	0.009–15.777	0.509
Smoking habit (yes/no)	1.144	0.090–14.570	0.917	1.890	0.109–10.222	0.999
Number of children	0.789	0.341–1.825	0.580	0.475	0.030–7.597	0.599
Group 2: Postmenopause						
WC (cm)	0.947	0.867–1.036	0.235	0.925	0.857–0.999	0.047
Glucose (mg/dL)	1.005	0.939–1.076	0.884	1.000	0.990–1.011	0.989
Triglycerides (mg/dL)	0.998	0.983–1.012	0.772	1.001	0.996–1.007	0.616
HDL (mg/dL)	0.993	0.943–1.047	0.807	1.000	0.944–1.059	0.990
Systolic blood pressure (mmHg)	1.035	0.964–1.111	0.337	0.990	0.946–1.036	0.990
Diastolic blood pressure (mmHg)	1.012	0.940–1.089	0.753	1.000	0.944–1.059	0.995
Age (years)	1.113	0.962–1.287	0.149	1.298	1.109–1.520	0.001
BMI normal (kg/m^2^)	1.399	0.111–17.565	0.795	2.387	0.319–17.855	0.397
BMI ≥ 25 kg/m^2^	0.083	0.007–1.023	0.083	1.784	0.494–6.448	0.377
Smoking habit (yes/no)	1.532	0.095–12.666	0.999	0.772	0.148–4.020	0.759
Number of children	1.619	0.953–2.750	0.075	0.674	0.456–0.997	0.048

Abbreviations: BMI: body mass index; HDL high-density lipoprotein; WC: waist circumference. OR: odds ratio, 95% CI: 95% confidence interval, significance at *p* < 0.05.

**Table 3:** Multivariate logistic regression analysis of low bone mineral density (osteopenia/osteoporosis) in the spine in 40–60-year-old women.

	No MetS			MetS		
	OR	95% CI	*p*	OR	95% CI	*p*
Group 1: Reproductive/Menopausal transition						
WC (cm)	0.989	0.902–1.084	0.811	0.790	0.635–0.982	0.034
Glucose (mg/dL)	1.023	0.958–1.093	0.490	0.988	0.930–1.049	0.692
Triglycerides (mg/dL)	1.005	0.991–1.020	0.468	0.997	0.987–1.006	0.505
HDL (mg/dL)	0.981	0.931–1.033	0.461	0.897	0.766–1.050	0.176
Systolic blood pressure (mmHg)	1.014	0.932–1.104	0.745	1.013	0.928–1.107	0.765
Diastolic blood pressure (mmHg)	0.952	0.854–1.061	0.369	0.955	0.854–1.070	0.429
Age (years)	1.246	1.016–1.528	0.035	1.309	1.013–1.690	0.039
BMI normal (kg/m^2^)	1.332	0.082–21.598	0.840	0.001	0.001–20.633	0.999
BMI ≥ 25 kg/m^2^	2.381	0.210–26.948	0.483	0.007	0.003–0.476	0.021
Smoking habit (yes/no)	3.354	0.264–42.529	0.350	0.005	0.001–0.509	0.025
Number of children	0.878	0.396–1.948	0.750	0.657	0.291–1.483	0.312
Group 2: Postmenopause						
WC (cm)	0.990	0.932–1.052	0.755	0.964	0.916–1.014	0.156
Glucose (mg/dL)	1.038	0.990–1.089	0.124	1.013	1.001–1.026	0.037
Triglycerides (mg/dL)	0.999	0.989–1.009	0.831	0.997	0.992–1.002	0.199
HDL (mg/dL)	1.024	0.984–1.067	0.245	1.084	1.030–1.140	0.002
Systolic blood pressure (mmHg)	1.012	0.959–1.067	0.667	0.981	0.947–1.017	0.307
Diastolic blood pressure (mmHg)	0.977	0.924–1.033	0.409	1.028	0.983–1.075	0.232
Age (years)	1.049	0.951–1.157	0.342	1.189	1.070–1.322	0.001
BMI normal (kg/m^2^)	1.847	0.257–13.262	0.542	2.037	0.298–13.950	0.468
BMI ≥ 25 kg/m^2^	1.309	0.284–6.028	0.730	1.282	0.453–3.632	0.640
Smoking habit (yes/no)	4.966	0.206–119.912	0.324	0.305	0.064–1.465	0.138
Number of children	1.247	0.864–1.798	0.238	0.611	0.431–0.867	0.006

Abbreviations: BMI: body mass index; HDL high-density lipoprotein, WC: waist circumference. OR: odds ratio, 95% CI: 95% confidence interval, significance at *p* < 0.05.

**Table 4:** Association between low bone mineral density (osteopenia/osteoporosis) in the dual femur and spine and components of MetS according to stages of reproductive aging ^a^.

	Group 1: Reproductive/Menopausal Transition (*n* = 146)			Group 2: Postmenopausal (*n* = 230)		
	OR	95% CI	*p*	OR	95% CI	*p*
Dual femur						
WC ≥ 80 cm	0.650	0.157–2.689	0.552	1.666	0.595–4.668	0.331
WC ≥ 88 cm	7.638	1.607–36.298	0.011	2.600	1.023–6.609	0.045
Triglycerides ≥ 150 mg/dL	1.200	0.271–5.321	0.810	0.708	0.336–1.494	0.365
HDL < 50 mg/dL	3.639	1.039–12.743	0.043	1.489	0.595–3.730	0.395
Blood pressure ≥ 130/85 mmHg or antihypertensive treatment	0.324	0.075–1.408	0.133	2.634	1.150–6.035	0.022
Fasting blood glucose ≥ 100 mg/dL or antidiabetic treatment	1.181	0.269–5.178	0.826	0.588	0.295–1.175	0.133
Spine (L1–L4)						
WC ≥ 80 cm	0.845	0.202–3.531	0.818	1.405	0.496–3.977	0.603
WC ≥ 88 cm	1.147	0.351–3.745	0.820	1.251	0.538–2.908	0.522
Triglycerides ≥ 150 mg/dL	0.946	0.289–3.099	0.927	1.112	0.577–2.144	0.751
HDL < 50 mg/dL	1.563	0.489–5.001	0.452	2.654	1.092–6.447	0.031
Blood pressure ≥130/85 mmHg or antihypertensive treatment	0.630	0.179–2.212	0.471	1.304	0.683–2.490	0.422
Fasting blood glucose ≥ 100 mg/dL or antidiabetic treatment	1.140	0.348–3.729	0.829	0.843	0.448–1.589	0.598

^a^ Adjusted for presence of metabolic syndrome, age, smoking habit, and number of children. Abbreviations: WC: waist circumference, HDL: high-density lipoprotein. Spine (L1–L4): spine at lumbar vertebra 1 to 4. OR: odds ratio, 95% CI: 95% confidence interval, significance at *p* < 0.05.

## Data Availability

There are restrictions on the availability of data for this trial, due to the signed consent agreements around data sharing, which only allow access to external researchers for studies following the project purposes. Requestors wishing to access the trial data used in this study can make a request to pep.tur@uib.es.
